# Palmitoylation of Voltage-Gated Ion Channels

**DOI:** 10.3390/ijms23169357

**Published:** 2022-08-19

**Authors:** Silvia Cassinelli, Carla Viñola-Renart, Anna Benavente-Garcia, María Navarro-Pérez, Jesusa Capera, Antonio Felipe

**Affiliations:** 1 Molecular Physiology Laboratory, Departament de Bioquímica i Biomedicina Molecular, Institut de Biomedicina (IBUB), Universitat de Barcelona, 08028 Barcelona, Spain; 2 Kennedy Institute of Rheumatology, University of Oxford, Oxford OX3 7FY, UK

**Keywords:** palmitoylation, voltage-gated ion channels, posttranslational regulation, diseases

## Abstract

Protein lipidation is one of the most common forms of posttranslational modification. This alteration couples different lipids, such as fatty acids, phospho- and glycolipids and sterols, to cellular proteins. Lipidation regulates different aspects of the protein’s physiology, including structure, stability and affinity for cellular membranes and protein–protein interactions. In this scenario, palmitoylation is the addition of long saturated fatty acid chains to amino acid residues of the proteins. The enzymes responsible for this modification are acyltransferases and thioesterases, which control the protein’s behavior by performing a series of acylation and deacylation cycles. These enzymes target a broad repertoire of substrates, including ion channels. Thus, protein palmitoylation exhibits a pleiotropic role by differential modulation of the trafficking, spatial organization and electrophysiological properties of ion channels. Considering voltage-gated ion channels (VGICs), dysregulation of lipidation of both the channels and the associated ancillary subunits correlates with the development of various diseases, such as cancer or mental disorders. Therefore, a major role for protein palmitoylation is currently emerging, affecting not only the dynamism and differential regulation of a moiety of cellular proteins but also linking to human health. Therefore, palmitoylation of VGIC, as well as related enzymes, constitutes a novel pharmacological tool for drug development to target related pathologies.

## 1. Introduction

Lipids are essential components of cell membranes that confine inner components. This compartmentalization also requires complex signaling pathways mediated by a wide range of cytosolic, transmembrane and membrane-associated proteins. These proteins are themselves regulated by the modification of specific groups in their amino acid chains [1]. This can influence many chemical and physiological mechanisms such as hydrophobicity or peptide electric charge. One of the most common protein modulations is lipidation. This process couples different lipids, such as fatty acids, phospho- and glycolipids and sterols, to proteins. Lipidation depends on different events regulating different aspects of protein physiology such as structure, stability, affinity for cellular membranes and protein–protein interactions. The lipid bond usually involves the N- or C-terminal domains of a target protein and requires amide, ether and ester binding to mostly glycine or cysteine residues [2,3]. To date, two main locations include this protein modification: (i) the lumen of the secretory pathway and (ii) the cytoplasm or at the cytoplasmic side of membranes. In this context, prenylation (thioether bond on cysteines), *N*-myristoylation (amide bond specific for glycines) and *S*-palmitoylation (cysteine thiols by thioester bond) generally take place in the cytoplasm [4,5]. *S*-palmitoylation is potentially reversible, and acyltransferases and thioesterases mediate this mechanism. These proteins regulate many cellular functions carrying out continuous acylation and deacetylation cycles. Because palmitoylation modulates a wide range of proteins including ion channels and alterations correlate with important diseases, such as cancer or mental disorders [6], this review summarizes the *S*-palmitoylation-dependent regulation of VGIC and related human maladies.

## 2. Protein Acylation

*S*-acylation consists of the posttranslational attachment of long saturated fatty acid chains to amino acid residues of soluble, transmembrane and peripheral membrane proteins. This process includes both monosaturated and polyunsaturated lipids. Considering the addition of palmitate and the amino acid involved, palmitoylation organizes into three different categories:

- *S*-palmitoylation or thioesterification is the major form of protein palmitoylation and consists of the addition of a palmitic acid (C16:0) to cysteines via a thioester bond [7].
- *O*-palmitoylation is the addition of palmitic (C16:0) or palmitoleic (C16:1) acids to serines or threonines by an oxyester bond [8,9,10].
- *N*-palmitoylation is the addition of a palmitic acid to N-terminal glycines, lysines or cysteines. Even though protein palmitoylation is potentially reversible, the N-type does not fit this rule and forms stable amide bonds [11,12,13].

Because the types of proteins undergoing palmitoylation are quite diverse, the function of this modification depends on the target. In general, palmitoylation increases the hydrophobicity of protein domains, facilitating their membrane association and stabilization, as well as contributing to protein–protein interactions and subcellular trafficking among membrane organelles and within specific membrane nanodomains [14,15]. For instance, the palmitoyl chain acts as a lipid anchor for various secreted signaling proteins such as the membrane fusion synaptosomal-associated protein 25 (SNAP25). In addition, palmitoylation controls the patterning of SNAP25 across the endoplasmic reticulum (ER) and the trans-Golgi network. In the case of Gα subunits of trimeric G proteins, palmitoylation mediates the phosphorylation-dependent internalization of the protein, ensuring correct trafficking from the ER to the Golgi and targeting lipid raft membrane nanodomains (LRs). Instead, the loss of the region containing palmitoylation sites in Ras GTPases inhibits H-RAS from moving out of LRs and thereby the correct activation of the Raf-MAPK cascade. [16,17,18,19,20]. Therefore, palmitoylation has pleiotropic effects on cell biogenesis.

Palmitoyl acyltransferase enzymes (zDHHC-PATs) are responsible for this modification. These proteins present a conserved Asp-His-His-Cys motif, which is necessary for catalysis. zDHHC-PATs are distributed in many cellular compartments, including the ER, Golgi apparatus and endosomes [21,22]. Thus, they increase *S*-palmitoylation heterogeneity by transporting proteins among different cellular compartments throughout subsequent cycles of acylation and deacylation. However, although the reaction involves the amino acid cysteine, no consensus sequence governs this mechanism [23,24]. The importance of protein palmitoylation is steadily emerging, not only for the dynamism and regulation of a wide range of proteins but also in several diseases, such as cancer, Huntington’s disease, long QT syndrome and a series of mental disorders, such as schizophrenia, Alzheimer’s disease and Parkinson’s disease [25,26,27,28]. Different methodologies have been developed for detecting interactions between proteins and lipids, comprising classical biochemical assays (acyl-biotin exchange (ABE)-palmitoylation assay), cellular radio-labeling by palmitate (^3^H-palmitate) visualized by SDS–PAGE, or mass spectrometry, cryo-electron microscopy (EM) mass spectrometry and super-resolution imaging techniques [29,30,31,32]. In this review, we concentrate on the *S*-palmitoylation of voltage-gated ion channels. In addition, terms such as thioesterification and *S*-acylation mostly refer to *S*-palmitoylation.

## 3. *S*-Palmitoylation of Ion Channels

Ion channels (IChs) participate in myriad physiological processes because they are expressed in all mammalian tissues such as in the nervous, muscular, glandular and immune systems. They generally associate to form either excitatory or inhibitory transmembrane complexes which allow ion fluxes across the cell membranes and organelles upon ligand binding or voltage changes. Thus, IChs are multifunctional proteins that work in distinct cellular compartments [33]. To date, evidence supports claims that *S*-palmitoylation may modulate different steps of the ICh life cycle, including membrane trafficking and spatial organization, as well as electrophysiological properties [34]. For instance, epithelial sodium channels (ENaCs), which belong to the ENaC/degenerin family, are responsible for amiloride sensitivity and the maintenance of Na^+^ and Li^+^ homeostasis. Because Na^+^ is the primary cation equilibrating the extracellular fluid volume, ENaCs are important in controlling this process, as well as blood pressure and airway surface fluid [35]. ENaCs are composed of three homologous α, β and γ subunits. However, only β and γ undergo *S*-palmitoylation at the junction between the N-terminal cytoplasmic domain and the first transmembrane segment of channels. By improving the interaction of ENaC cytoplasmic domains with the plasma membrane, *S*-palmitoylation results in conformational changes that increase the open state of the targeted channel. Even though palmitoylation enhances the membrane affinity for some channel regions, it does not participate in surface expression or channel membrane trafficking [36,37].

In addition, the α-amino-3-hydroxy-5-methyl-4-isoxazolepropionic acid receptors (AMPARs), which are a series of ligand-gated cation channels resulting from different assemblies of four GluR1-4 subunits, are palmitoylated. These subunits are strongly conserved within the transmembrane and extracellular N-terminal domains, while the intracellular C-terminus diverges. AMPARs are important modulators of the excitatory postsynaptic currents in the nervous system. Both the transmembrane 2 (TMD2) and the C-terminal domains undergo thioesterification but exhibit distinct effects. The *S*-palmitoylation of TMD2, by GODZ acyltransferase (i.e., zDHHC3), mainly occurs in the Golgi apparatus, impairing AMPA surface expression and triggering Golgi retention of GluRs. However, N-terminal depalmitoylation restores the channel’s physiology. Interestingly, the addition of palmitate to the C-terminal cysteines impairs the interaction between GluR1 and the 4.1N protein, which is responsible for AMPA internalization and triggering the opposite regulation of AMPA channels [38,39].

The targets of palmitoylation are quite diverse, and sometimes accessory peptides, rather than the conducting subunits, undergo modification. For instance, Barttin, the modulatory subunit of human chloride channels (ClC-Ks), is expressed in the kidney and inner ear, enhancing the membrane traffic and the activation state of the channels. Deficient Barttin palmitoylation reduces the channel current amplitude but does not alter the presence of ClC-K/Barttin complexes at the cell surface. This evidence supports the idea that *S*-palmitoylation of Barttin affects ClC-Ks activation by causing structural changes at the plasma membrane [40,41]. In the case of the mammalian water channel aquaporine 4 (AQP4), *S*-palmitoylation occurs on the N-terminal cysteines, lying next to hydrophobic residues. Although there are no specific consensus sequences, this posttranslational modulation is highly conserved in vertebrates for AQP4 [42]. AQP4 is mainly expressed in the brain where, upon association, it gives rise to typical square-array clusters. Thus, AQP4 drives bidirectional water flow across the blood–brain barrier. Palmitoylation controls AQP4 spatial organization by inhibiting square formation and thereby impeding the constitution of a functional channel [43]. Further regulation governed by this modification is observed in sodium–calcium exchanger 1 (NCX1), playing a dual role. On the one hand, palmitoylation alters protein–protein interaction mechanisms by promoting NCX1 dimerization, which in turn prevents the interaction with endogenous exchange inhibitory peptide (XIP). This phenomenon leads to lower protein inactivation. On the other hand, palmitoylation affects the channel affinity for LRs, triggering an increase in transmembrane Ca^2+^ flux [44]. Because NCX1 is crucial in the regulation of Ca^2+^ transport, upon control of transmembrane Na^+^ gradients, altering palmitoylation may provide benefits in the study of the immunological consequences of elevated cytosolic Ca^2+^ levels [45]. Therefore, palmitoylation undertakes central functions in ion channel regulation. However, considering the wide functional spectrum of ion channels, scarce information is available about this posttranslational modification in voltage-gated ion channels (VGICs).

### 3.1. Palmitoylation of Voltage-Gated Ion Channels

VGICs are integral membrane proteins that share similar architectures and respond to electrical stimuli controlled by the voltage along cellular membranes. By opening and closing the pore, VGICs modulate ion fluxes, playing a crucial role in electrical signaling and cell homeostasis. A Na^+^ channel from the eel electroplax and a K^+^ channel from the *Shaker* mutant of *Drosophila melanogaster* were the first purified channels. These discoveries paved the way for the cloning of numerous VGIC channels in different species. Based on ion conductance, these proteins are divided into voltage-gated Na (Nav), Ca^2+^ (Cav) and K^+^ (Kv) channels [46]. Functional Nav and Cav are structured in an α-subunit composed of four homologous repeats, each presenting six transmembrane segments S1–S6. Kv channels consist of only one domain, homologous to the four domains of Na and Ca^2+^ channels, but form tetramers. The most conserved features of those channels are the voltage sensor, which resides at the S4 domain, and the selectivity filter placed at the pore-region between the S5 and S6 domains. All of these channels may act in association with ancillary β subunits. While Nav channels are crucial for the initiation of action potentials in excitable cells and Cav activate upon depolarization events and calcium entry, Kv channels are responsible for maintaining the resting-membrane potential of cells. Briefly, a polarized cell membrane consists of a negative voltage at the inner cell side with respect to a positive voltage at the exterior. In this situation, Kv channels are in the closed state. When a depolarization event occurs, they rapidly open driving ions through passive transport along the electrochemical gradient. In this way, Kv brings the cellular membrane potential back to the resting state. Since VGICs are fundamental for many cellular processes, posttranslational regulation is crucial to the final physiological fate of these proteins. In this scenario, *S*-palmitoylation represents the most common form of VGIC acylation affecting different features of their biogenesis [47,48,49].

#### 3.1.1. Voltage-Gated Sodium Channels

Voltage-gated sodium channels (Nav) are heterotrimeric proteins composed of one pore-forming α and two accessory β subunits that are essential for the generation and propagation of Na^+^-dependent action potentials. Nav presents a characteristic 260 kDa α subunit, forming the conducting pore, in association with accessory β subunits through noncovalent bonds. However, α subunits alone may recapitulate functional channels. Palmitoylation occurs in the early stages of Nav channel biosynthesis. Very frequently, there is crosstalk with other posttranslational modifications. Thus, the RII subtype can be palmitoylated, glycosylated and sulfated simultaneously [50,51]. Because Nav channels drive fundamental signaling processes during synaptic events in neurons, posttranslational regulation is crucial for correct channel functioning. Palmitoylation can affect different regions of the Nav structure, triggering different consequences. For example, Nav1.2 palmitoylation strongly affects channel pharmacology by altering the affinity for the tarantula toxin PaurTx3 at the II domain, which is responsible for the voltage sensitivity and inward flux of sodium [50,52]. Cardiac Nav1.5, which is responsible for the propagation of cardiac electric activity, is palmitoylated on Cys981, and four more cysteine residues are placed at the cytoplasmic loop between the II and III domains of the channel. Similar to Nav1.2, thioesterification of this specific region affects the voltage-dependence properties of Nav1.5. Moreover, augmenting the membrane affinity of the II–III linker causes a shift in the voltage sensor domain (III), which is crucial for channel inactivation [53]. Nav1.6 is also palmitoylated at three putative sites: (i) Cys1169-1170, highly conserved in human Nav channels, and (ii) Cys1978, exclusive in Nav1.6. Mutation of these conserved residues, concomitantly with the impossibility of undergoing palmitoylation, causes a hyperpolarization shift, slowing channel recovery from the inactivation state. *S*-palmitoylation of Cys1978 alters the channel current density but not the voltage dependence, suggesting that thioesterification of this specific residue controls the surface expression of Nav1.6 [54].

#### 3.1.2. Large Conductance Calcium-Activated Potassium (BK) Channels

**Figure 1:**
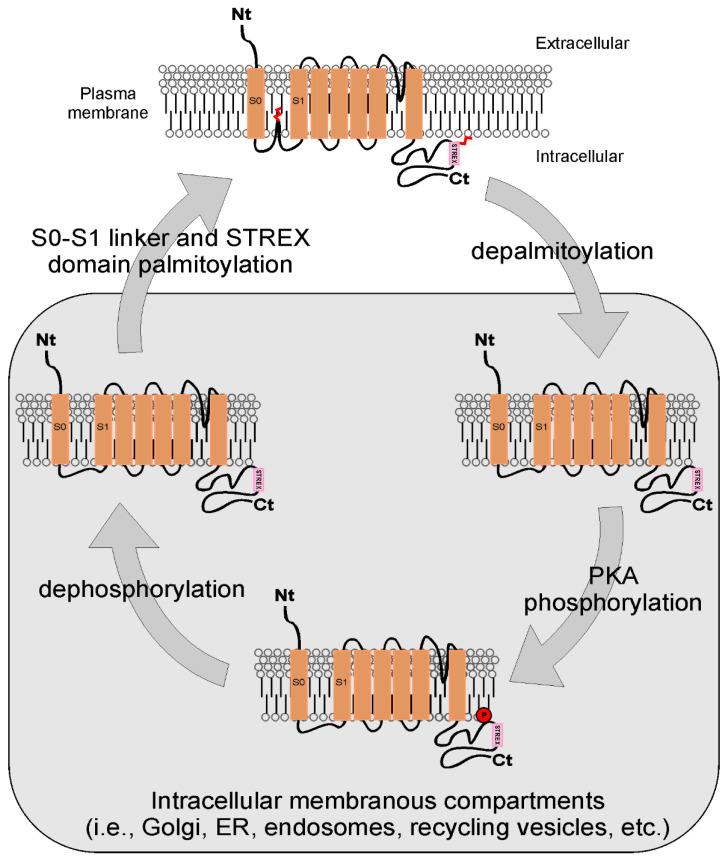
BK channel palmitoylation cycle. BK channels undergo *S*-palmitoylation on the *N*-terminal S0-S1 link and on the C-terminal STREX domain. The S0-S1 loop and STREX motif palmitoylation strongly promotes the surface expression. Depalmitoylation of the STREX motif exposes BK channel to PKA phosphorylation and decreases membrane abundance, shifting the channel from plasma membrane to intracellular membranous compartments. Dephosphorylation facilitates a new palmitoylation process and the plasma membrane surface expression.

Large conductance calcium- and voltage-gated potassium (BK) channels are important regulators of membrane excitability in the central and peripheral nervous systems, along with the vascular and endocrine systems. BK repolarizes cellular membranes after depolarization and a concomitant increment of cytosolic Ca^2+^. Higher levels of intracellular Ca^2+^ trigger a signaling pathway shifting open voltage-gated Ca^2+^ channels to a closed conformation [55]. BK channels are palmitoylated at both the N- and C-termini. More particularly, the S0-S1 loop (S0-S1 linker) at the N-terminal domain presents three palmitoylation sites (Cys53, 54 and 56). Because the addition of palmitate increases the protein hydrophobicity, mutations of these residues lead to a drastic reduction in the channel’s membrane expression and insertion [56]. Two different palmitoyl acyltransferases, zDHHC22 and zDHHC23, are responsible for the S0-S1 linker acylation with no additive effects, suggesting that they work within the same pathway. On the other hand, the C-terminal domain is palmitoylated on cysteine residues composing the STREX complex. In this context, thioesterification of distinct regions of the same BK molecule leads to similar effects. Indeed, the abolishment of *S*-palmitoylation results in lower membrane expression of BK channels without affecting their conformation and electrophysiology. Moreover, a polybasic domain (Lys623 to Arg643) in the region upstream of palmitoylated cysteines can be phosphorylated at Ser636. PKA phosphorylation of Ser636 controls the membrane insertion of the STREX domain and, in turn, *S*-palmitoylation, which is driven by zDHHC3,5,7,9,17 at the cell surface compartment [57]. Therefore, the modulation of BK inhibition is the result of cooperation between palmitoylation and PKA-dependent phosphorylation of the STREX complex (Figure 1). The crosstalk with other posttranslational modifications is an additional feature of the dynamism and heterogeneity of protein lipidation [58,59].

#### 3.1.3. Voltage-Dependent Potassium Channels

Voltage-dependent potassium channels (Kv) represent a large family of transmembrane proteins with more than 40 members, divided into 12 subfamilies only in humans, that are evolutionarily conserved and extensively expressed in multiple tissues, including excitable and non-excitable cells. For that reason, Kv functions are important in a plethora of physiological processes. In general, during an action potential Kv channels conduct K^+^ ions out of the cell, returning the membrane potential to the resting state. In the nervous, cardiovascular and muscular systems, Kv contributes to the modulation of waveform and action potential responses [60]. In non-excitable cells, such as the immune system, Kv controls cell volume, activation, proliferation, migration and apoptosis. Kv channels are posttranslationally regulated by a wide range of modifications, including phosphorylation, N-glycosylation, ubiquitination, redox modifications and lipidation [61]. In this context, upon *S*-acylation, the majority of Kv channels are palmitoylated and belong to the *Shaker* subfamily. For instance, Kv1.1, Kv1.2 and Kv1.4 are palmitoylated by zDHHC14 in neurons [62]. In particular, Kv1.1 is covalently palmitoylated at Cys243 in Sf9 cells, and mutations of this residue reduce the macroscopic current and alter the activation of the channel, which will activate at a more electronegative voltage. Finally, the channel needs more depolarized potential to be inactivated in the absence of palmitoylation. Notably, mutations of other cysteines trigger no significant changes in Kv1.1 currents. Therefore, C243 alone is responsible for Kv1.1 acylation, clearly affecting the channel electrophysiology [63,64]. Similarly, although Kv1.5 can be palmitoylated in both the COOH and the NH2 terminus, only a C-terminal cysteine is responsible for channel thioesterification. The addition of palmitate at this level triggers the intracellular confinement of the channel. Moreover, palmitoylated Kv1.5 redistributes into different intracellular compartments, skipping channel degradation. Thus, Kv1.5 C-terminal *S*-palmitoylation impairs the proteasome-dependent degradation of the channel. Because the degradation rate of palmitoylated Kv1.5 channels is lower, their surface expression increases, and K^+^ currents are generated [64,65].

The abovementioned evidence highlights a palmitoylation-dependent pleiotropic role in the regulation of VGIC. This PTM triggers different effects depending on numerous factors, such as the target protein, the specific domain, the cellular compartment and the catalytic enzyme. All these possibilities suggest that palmitoylation is an important mechanism for proper VGIC biogenesis and function.

## 4. Palmitoylation of Channelosomes

**Figure 2:**
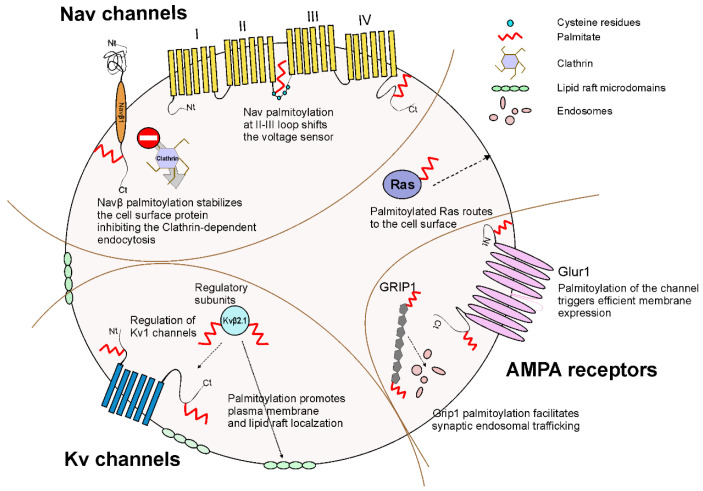
Schematic representation of multiple palmitoylation effects on VGIC and regulatory subunits. AMPA receptors are also shown. Palmitoylation may affect traffic and membrane stabilization, but it can also modulate other cellular mechanisms such as endocytosis and recycling. Alterations of the electrophysiological properties have been also documented.

The channelosome is a term that usually defines the pore-conducting complex associated with a number of ancillary proteins that modulate channel function. Most of the time, palmitoylation affects these accessory proteins within different channelosomes, which in turn fine-tunes ion channel physiology. Therefore, this modification may affect VGIC properties directly or indirectly by influencing either the conducting subunits or the modulatory proteins within the VGIC regulatory machinery (Figure 2).

### 4.1. Palmitoylation Affects Membrane Association and Protein Stability

As mentioned above, palmitoyl acyltransferases and thioesterases mediate the palmitoylation of soluble and transmembrane proteins, influencing their trafficking and membrane localization. In the nervous system, glutamate receptor proteins 1b and 2b (GRIP1b/2b) are palmitoylated by zDHHC5/8 through the presence of several PDZ domains, which mediate heterophilic protein–protein interactions. In this context, GRIP1b represents a specific link between endosomes and microtubule motors [66,67]. GRIP1b *S*-palmitoylation facilitates synaptic vesicle trafficking in neurons, accelerating AMPA receptor recycling [68,69]. Moreover, the zDHHC5 cycling between the plasma membrane and endosomes of dendrites, contributes to the stability of AMPA channels at the postsynaptic membrane [70]. On the other hand, Nav β1/2 are non-pore-forming subunits associated with Nav channels. These proteins belong to the Ig subfamily of cell adhesion molecules due to the presence of V-type immunoglobulin domains. Navβ1/2, expressed in many tissues, including the brain and heart, regulate membrane trafficking, gating and kinetics of Nav channels. In addition, Navβ1/2 subunits also modulate potassium currents, cell–cell and cell–matrix adhesion, cell migration and intracellular calcium signaling pathways [71]. The Navβ1 subunit undergoes *S*-palmitoylation at Cys162. Addition of palmitate enhances the β1 membrane association and stabilizes the polypeptide at this compartment while reducing the clathrin-mediated endocytosis of the protein [72]. The postsynaptic density-95 (PSD-95) protein, a member of the membrane-associated guanylate kinase (MAGUK) family, is a PDZ domain-containing polypeptide. PSD-95 contains three postsynaptic density-95 disc-large and zonulin-1 (PDZ) motifs, close to an SH3 domain [73,74]. PDZs are variable length linkers abundant in eukaryotic genomes that play main roles in assisting the formation of signaling complexes, clustering membrane receptors, and cell polarity maintenance. Various ion channels, including N-methyl-D-aspartate (NMDA) receptor subunits, inwardly rectifying (Kir) K^+^ and *Shaker* channels, interact with PSD-95 [75,76]. The binding with PSD-95 involves a tSXV consensus sequence placed at the channel C-terminal domain and the two first PDZ domains of the kinase [77]. In this scenario, PSD-95 interacts with Kv1.4, causing the aggregation of both proteins at the cell surface. *S*-palmitoylation of PSD-95 at N-terminal Cys3–5 residues is necessary for proper function during channel clustering. Blockage of protein thioesterification dysregulates perinuclear trafficking, postsynaptic targeting and ion channel clustering activity [78].

Further examples of palmytoilation of ancillary subunits include Kvβ proteins. These soluble polypeptides are present in many Kv channelosomes, such as Kv1.3 and Kv1.5, conferring diversity to those channels. Kvβs tetramerize and bind 1:1 to tetrameric Kvα subunits [79,80,81]. Kvβs may affect not only the overall kinetics but also the biosynthesis of α-subunits [82,83]. Although both Kvβ1 and Kvβ2 members are palmitoylated, only the latter partially targets specific plasma membrane lipid raft nanodomains. *S*-palmitoylation of distal cysteines 301 and 311 drives Kvβ2.1 to this location in the absence of α subunits, and palmitoylated Kvβ2.1 targets the immunological synapse (IS). This evidence points to this mechanism as an important regulator during the immunological response [79]. Furthermore, Kv4 is crucial for the integration of synaptic inputs and for action potential organization not only in neurons but also in cardiomyocytes. EF hand-containing Ca^2+^-binding proteins (KChIPs) regulate channel expression and spatial location. They are encoded by at least four genes, KChIP1–4, consisting of a conserved core region and a diverse N-terminus. Similar to Kvβs, soluble KChIP peptides undergo palmitoylation and traffic to the cell surface in complex with the α subunits. This posttranslational event deeply affects the modulation of the associated channel. Thus, Kv4.3 exhibits higher current densities in the presence of palmitoylated KChIP2 concomitantly with an elevated number of channels at the cell membrane [84]. Simultaneously, palmitate attachment reduces the cytoplasmic mobility of KChIP2, thereby stabilizing the protein in the cytosol. However, when necessary, palmitoylation also drives KChIP2 distribution between the nucleus and plasma membrane, promoting channel surface trafficking. In summary, thioesterification of auxiliary subunits may result in double regulation. First, β subunit homeostasis is directly affected by altering cellular localization. Second, palmitoylation affects channel function by spatial reorganization [85].

Interactions with ancillary polypeptides include β-secretase 1 (BACE1). *S*-palmitoylation of four C-terminal cysteines (Cys474, 478, 482, 485) influences the protease activity responsible for amyloid precursor protein (APP) cleavage. BACE1 functions in membrane rafts where it drives APP in the amyloidogenic direction, causing β-amyloid (Aβ) production. When APPs are outside of raft domains, they are preferentially cleaved by nonamyloidogenic α-secretase. Because BACE1 uses palmitoylation for this specific membrane anchoring, the modification is critical not only for its cellular localization but also for pathologic Aβ generation [86]. BACE1 regulates diverse ion channels such as the M-type K^+^ channels (KCNQ1–5) also named Kv7 [87]. Kv7 forms heteromeric channels that are widely expressed in the central nervous system, especially Kv7.2 and Kv7.3. Kv7 channels are critical for the transduction of sympathetic stimuli, for the muffling of excessive neuronal excitability and for controlling neuronal activation by reducing excitatory postsynaptic potentials. Kv7 channels localize to membrane rafts upon interaction with palmitoylated BACE1, leading to a new gating rearrangement [88,89]. Finally, palmitoylation of the regulatory β4 subunit of BK channels at Cys193 promotes exit from the ER and increases surface expression, representing an additional mechanism governing BK channel functions [90]. Therefore, palmitoylation of ancillary peptides in channelosomes either directly or indirectly influences the functional properties of the channels.

### 4.2. Palmitoylation Affects Electrophysiological Properties

Unlike the majority of VGICs, the high voltage-activated (HVA) Cav1 and Cav2 Ca^2+^ channels may act exclusively upon association with an auxiliary β subunit (Cavβs). The complex traffics to the plasma membrane to function. The Cav1.3 channel is inhibited by arachidonic acid (AA), but the presence of palmitoylated β2a peptides impairs this blockage. Both palmitate and β2a compete with AA, avoiding channel inhibition and with the PI(4,5)P2-phosphatidylinositol 4,5-bisphosphate (PIP_2_) binding site, protecting the channel from PIP_2_-mediated depletion. However, thioesterification is not required for the membrane anchoring of α subunits [91]. In α1C and α1E neuronal Ca^2+^ channels, the β2a subunit facilitates no channel prepulsiveness, which consists of a strong depolarization input leading to an increment of pore opening. Moreover, it inhibits α1E voltage-dependent inactivation. This tight regulation only occurs when β2a is palmitoylated. Thus, altering palmitoylation sites (Cys3, 4) or using pharmacological inhibition does not trigger an increase in the steady-state inactivation of Ca^2+^ channels. Thioesterification of β2a regulates both the accessory subunit and the associated α subunit, further modulating the palmitoylation of other β subunits in the complex [92,93,94].

ATP-sensitive K^+^ (KATP) channels are another example. These channels are formed by four inward rectifier K^+^ (Kir6.x) channel subunits, which present two members (Kir6.1 and Kir6.2) and four sulfonylurea receptor (SURx) subunits, also presenting two members (SUR1 and SUR2). Kir channels are sensitive to the ADP:ATP ratio. KATPs open in high ADP, coupling intracellular energy metabolism to membrane excitability. *S*-palmitoylation of Kir6.2, occurring on Cys166, activates the channel, leading to an increment in the open state by altering the dwell time, but neither traffic nor surface expression induces an overall augmentation of channel activity [95].

## 5. Interplay with Other Posttranslational Modifications

As mentioned above, most of the α subunits of VGIC exhibit crosstalk between palmitoylation and different posttranslational modifications (PTMs).

### 5.1. Phosphorylation

Reversible phosphorylation represents one of the most abundant posttranslational regulatory mechanisms. This process, catalyzed by protein kinases, links a phosphate group (PO_4_) to a polar R moiety of different amino acids. Similar to palmitoylation, this PTM shifts protein hydrophobicity toward a polar hydrophilic status, favoring conformational changes during interaction with other cellular partners. Palmitoylation, thereby, can be interpreted as the lipid version of protein phosphorylation. Phosphorylation affects a high number of VGICs and receptors. In ionotropic glutamate receptors, the palmitoylation of Glur6 reduces channel phosphorylation by PKC but inhibits PKC-mediated phosphorylation of Glur1. In both channels, the palmitoylated cysteine is contiguous to the consensus phosphorylation site [96]. Nevertheless, the fact that two palmitoylable and phosphorylable residues are in close proximity does not necessarily mean that the modifications must cooperate. Thus, TRPP3, a transient receptor potential (TRP) channel, initiates signal transduction pathways by altering membrane potentials or intracellular calcium levels. TRPP3 undergoes *S*-palmitoylation at the N-terminal residue Cys38 and phosphorylation at the next residue Thr39. However, the mechanisms regulate TRPP3 functions independently [97].

Palmitoylation of STREX alters PKA-dependent phosphorylation in the abovementioned BK channels (see Section 3.1.2). Specifically, BK proteins present a conserved C-terminal phosphorylation motif that, when phosphorylated, induces channel activation. In this scenario, the membrane insertion of the STREX motif at the channel C-terminus generates a new PKA phosphorylation site, with an opposite inhibiting effect (Figure 1). Evidently, the dissociation of the STREX domain from the plasma membrane prevents PKA inhibition. Because palmitoylation of STREX drives its membrane inclusion, the mechanism would be an antagonist to the channel inhibition mediated by PKA. Thus, the palmitoylation of the same subunit in a phosphorylated channel is crucial for gating properties [98]. The β2-adrenergic receptor (β2AR), a G protein-coupled receptor (GPCR), also undergoes both phosphorylation and *S*-palmitoylation. zDHHC9/14/18 acyltransferases mediate the palmitoylation of Cys265, but this process strictly requires two other conditions: (i) the PKA-mediated phosphorylation of Ser261/S262 residues and (ii) β2AR internalization. Both PTMs drive the trafficking of the receptor to the Golgi apparatus, where zDHHC9/14/18 resides [99,100,101].

### 5.2. N-myristoylation and Polyisoprenilation

*N*-myristoylation corresponds to the addition of a myristate (14-carbon fatty acid) to the N-terminal Gly, or in some cases to Lys, of a protein through a covalent amide bond. Unlike phosphorylation and palmitoylation, this mechanism is not reversible and occurs very early during protein biosynthesis in either co- or post-translational stages. Several α subunits of heterotrimeric G proteins, as well as distinct members of the Src family, are influenced by protein myristoylation. A consensus motif for dual acylation (Met-Gly-Cys) is situated within their N-terminal Src-homology-4 (SH4) domain. The *N*-myristoylation of Gly2 of Gαo-α subunit of heterotrimeric Go proteins is a necessary condition for the *S*-palmitoylation of Cys3 because the presence of myristate enhances the accessibility of zDHHCs for membrane binding. This evidence is interesting because it suggests possible interplay among *N*-myristoylation and/or *S*-palmitoylation with phosphorylation [102]. As mentioned above, the depalmitoylation of STREX motif of BK channels exposes the Ser3 residue (placed just upstream to the STREX palmitoylation site) and this allows the PKA-mediated inhibition of the channel. In addition, a cooperation between palmitoylation and polyisoprenylation has been documented in Ras proteins, which regulate many ion channels, including Nav channels [103,104]. Prenyltransferases, which are required for polyisoprene lipid attachment, produce stable thioether bonds with cysteine residues. This interplay triggers protein maturation, which is developed in two steps. Initially, Ras proteins, guided up to the interface between the membrane and cytosol, become substrates for acyltransferases [105,106]. Finally, Ras undergoes palmitoylation at Cys188. For instance, p21-Ras, polyisoprenylated at Cys186, is converted in an intermediate form (c-p21), which mostly resides at the cytoplasm interface because it is still not palmitoylated (step-I). Next, protein thioesterification allows conversion into the mature membrane-associated form (m-p21) of the protein (step II). Instead, the p21^K-rasB^ isoform, which lacks upstream Cys188 residues, cannot be palmitoylated. The final fate is that polyisoprenylated but non-palmitoylated Ras proteins are biologically active, but weakly associate with cell membranes. Palmitoylation increases the intensity of this binding. Therefore, *S*-palmitoylation either drives polyisoprenylated Ras to specific membrane compartments or modulates Ras activity by controlling the amount of the protein at the cell surface [107,108,109].

## 6. VGIC Palmitoylation and Diseases

### 6.1. Palmitoylation in Diabetes 

Diabetes is generally characterized by gradual apoptotic β cell death, which leads to a reduced β-cell density in the endocrine pancreas. This occurs because of an increase in blood glucose levels and the consequent pancreatic-β cell response, which consists of insulin release. This process, when protracted up to chronic levels, alters Ca^2+^ homeostasis and generates cellular toxicity, providing favorable conditions for hyperglycemia development and diabetes progression [110]. Ca^2+^ fluxes are governed by Cav channels, which are in turn modulated by association with Cavβ ancillary subunits. Since these complexes manage the maintenance of calcium homeostasis, they are important regulators during the compensation of insulin resistance, behind the chronic increment of blood glucose thresholds. Evidence shows that palmitoylation of Cavβ2a stabilizes the tethering of the pore-forming major voltage-gated Ca^2+^ channel subunit α1 in the plasma membrane. This phenomenon triggers an increase in Ca^2+^ concentrations in insulin-secreting β-cells. Consequently, overexpression of palmitoylated Cavβ2a induces apoptotic cell death. Type 2 diabetes, for example, involves both the alteration of insulin effects in peripheral tissues and the impairment of glucose-stimulated insulin secretion (GSIS). In this context, palmitate addition potentiates GSIS by activating voltage-sensitive L-type Ca^2+^ channels. The increment of intracellular calcium concentrations also leads to an augmentation of the readily releasable pool of granules (RRP) in the blood circulation, which consecutively causes the exocytosis of channels and high insulin secretion. Keeping all this in mind, controlling the palmitate addition may be crucial in the rescue of overactivated pancreatic β cells, as well as providing a new way to handle type 2 diabetes progression [111].

### 6.2. Palmitoylation and BK/Kv Channel-Associated Disorders

K^+^ channels play key roles in cell proliferation, division and migration and are therefore critical in the biology of human cancer. BK channels maintain the electrochemical conditions required for cell division and migration. Palmitoylation tightly regulates both α and β subunits, and in this context, the addition of palmitate may become crucial for the cell homeostasis equilibrium. Some studies propose a vaccine, considering the prolongation of BK channel activation by palmitoylation. Thus, the injection of rats with T9-C2 glioma cells, which exhibit prolonged activation of the BK channels, would cause a cytosolic ROS production leading to cell swelling, formation of vacuoles and cell death in gliomas. In fact, prolonged BK channel activation kills T9 cells and thereby these cells can be used as a prophylactic vaccine in rats. This would create a new cell-death pathway to rescue physiological conditions required for normal cell proliferation [112,113,114]. KCNQ1 (Kv7.1) dysfunction causes long QT syndrome progression. Alterations in Kv7.1 activity are also related to sudden infant death syndrome, congenital deafness and familial atrial fibrillation. Because the lipid bilayer is crucial in maintaining the relative orientation of membrane proteins, understanding the structural properties of Kv7.1 in a lipidic environment may be key for the management of pathological consequences [115]. Mutations in K^+^ channels and their related accessory subunits also participate in different human epileptic phenotypes. For instance, both Cav and Kv channel dysregulation may result in CLN1 disease, an inherited neurological disorder of early childhood characterized by epileptic seizures and premature death. Studies in the human neuroblastoma-derived (SH-SY5Y) cell line show that CLN1 overexpression induces the selective suppression of Cav and Kv currents, triggering the reorganization of cellular electrophysiological properties. In fact, the expression of Kv11, Kv12 and Kv7 channels is strongly reduced upon CLN1 transfection. Because defects in the CLN1 gene (coding for palmitoyl-protein thioesterase 1 (PPT1)) are associated with CLN1 disease, the authors claim that PPT1 may have a protective role against excitotoxicity in in vivo-induced status epilepticus [116]. Therefore, targeting altered currents and related VGICs upon CLN1 overexpression may yield a better understanding of the pathophysiology of epilepsy events in CLN1 disease.

### 6.3. Alzheimer’s and Cystic Fibrosis Diseases

Alzheimer’s disease is characterized by the interaction between KChIP proteins and presenilin, controlling ER calcium signaling and apoptosis. Complex formation highly depends on cellular Ca^2+^ concentrations. Presenilins (Ps), part of the γ-secretase enzyme complex, cleave the amyloid precursor protein (APP) to produce βAmyloid (Aβ) peptides [117]. This process, when dysregulated, causes Alzheimer’s disease progression. The interaction between KChIP3 (calsenilin) and PS1 is highly Ca^2+^ dependent, and KChIP3 dimerizes by binding two presenilin molecules. Therefore, the KChIP3 and PS1/PS2 clusters are essential in both APP γ-cleavage and ER Ca^2+^ release, which are the main features of Alzheimer’s disease. Several KChIP members reach the plasma membrane and modulate the activation of Kv4 channels upon palmitoylation. In this scenario, palmitoylation also increases BACE1 lipid raft localization and BACE1-mediated cleavage of APP at these domains. Thus, APP palmitoylation enhances the amyloidogenic pathway responsible for Aβ production. Because palmitoyl acyltransferase inhibitors, such as cerulenin, decrease Aβ production, the pharmacological hindrance of APP palmitoylation might prevent Alzheimer’s evolution [118]. Finally, the cystic fibrosis-transmembrane conductance regulator (CFTR), an ion channel transporting chloride and bicarbonate across the apical surfaces of epithelia, is involved in cystic fibrosis. The inhibition of palmitoylation by 2-bromopalmitate (2-BP) impairs the biogenesis of CFTR, resulting in lower chloride transport. The prevention of palmitate addition at Cys524 triggers destabilization and premature degradation. zDHHC7 acyltransferase stabilizes the protein in the Golgi apparatus. Therefore, PATs and PPTs should be considered potential targets for CFTR rescue [119].

### 6.4. Mitochondrial Activity and Cancer Development

Mitochondria physiology fine-tunes the metabolism of cancer disease. In the cytosol, mitochondrial-associated membranes (MAMs) link the ER and mitochondria [120]. These contact points represent fundamental modulators of Ca^2+^ flow across the two compartments, which is fateful for correct mitochondrial functioning and ATP production. MAMs are important platforms for the localization of distinct types of proteins, and palmitoylation is the major mechanism responsible for protein MAM trafficking. For instance, the ER-localized thioredoxin-related transmembrane protein (TMX), which reaches MAMs in a palmitoylation-dependent manner, impairs the process and strongly reduces the protein level in MAMs [121,122]. On the other hand, cytoskeleton-associated protein 4 (CKAP4) requires *S*-palmitoylation to accomplish a proper role in cellular respiration. CKAP4 is a substrate of ZDHHC2, which forms a functional complex with voltage-dependent anion-selective channel protein 2 (VDAC2) localized to the outer mitochondrial membrane. Complex formation is dependent on CKAP4 palmitoylation. Mutations of the palmitoylation sites lead to the prevention of VDAC2 binding, compromising mitochondrial function. Therefore, palmitoylation can directly influence mitochondrial protein function. Moreover, palmitate synthesis, performed by FASN, mediates the palmitoylation of the mitochondrial epidermal growth factor receptor (mtEGFR) in mtEGFR-positive prostate and breast cancer cell lines. Collectively, these findings illustrate that palmitoylated CKAP4 in the ER regulates mitochondrial functions via interaction with VDAC2. Because glycolysis supports neoplastic cell proliferation and mitochondrial oxidative phosphorylation requires CKAP4, palmitoylation is decisive in the control of cancer cell proliferation and mitochondrial diseases [123].

## 7. Concluding Remarks

Ever-growing evidence provides a better understanding of palmitoylation and the consequent regulation of proteins in innate and adaptive immune responses, as well as many physiological and related pathologies. In this review, we provide updated information that clearly demonstrates the importance of this posttranslational modulation in VGIC biology (Table 1 and Table 2). Even though we manage extensive information about this mechanism and its consequent effects on targeted proteins, we still must move forward in the formulation of specific clinical drugs for fine-tuning protein palmitoylation. Therefore, with the findings summarized in this review we seek to merge novel information about lipidation on the role of VGIC and its implications in human diseases, providing innovative pharmacological targets to address associated disorders.

## Figures and Tables

**Table 1:** Palmitoylated channels and correlated disorders.

Channel Family	Channel	Palmitoylated Residue	Correlated Diseases	Reference
Epithelial sodium channels	ENaCs			[34,35,36]
ATP-sensitive potassium channels	Kir6.2	Cys166		[95]
Transient receptor potential (TRP) channels	TRPP3	Cys38		[97]
Chloride channels	CFTR	Cys524	Cystic fibrosis	[119]
Voltage-gated calcium channels	Cav1, 2			[93]
Large conductance calcium-activated potassium channels	BK	Cys53,54,56 STREX	Cancer CLN1 disease	[56,57] [116]
Sodium-calcium exchangers	NCX1			[42]
Voltage-gated potassium channels	Kv1.1 Kv1.2 Kv1.4 Kv1.5	Cys243		[63,64] [62] [62] [64,65]
Voltage-gated sodium channels	Nav1.2 Nav1.5 Nav1.6	Cys981 Cys1169-1170 Cys1978		[50,55] [50,55] [50,55]
Aquaporins	AQ4	Cys609		[42,43]
GluR1-4	AMPARs			[38,39]

**Table 2:** Palmitoylated regulatory subunits and ancillary proteins and correlated disorders.

Protein Family	Regulatory Subunit/Ancillary proteins	Palmitoylated Residue	Correlated Diseases	Reference
Glutamate receptor-interacting proteins	GRIP1b/2b			[66,67]
Clc-k accessory subunit, Barttin, family	BARTTIN			[40,41]
Cytoskeleton-associated protein 4 family	CKAP4		Cancer Mitochondria disorders	[122]
Voltage-gated sodium channels	Navβ1	Cys162		[72]
Voltage-gated calcium channels	Cavβ2a	Cys3, 4	Diabetes	[92,94,110]
Voltage-gated potassium channels	Kvβ2.1	Cys51, 212, 248, 301, 311		[79]
			Alzheimer’s	
Kv channel-interacting proteins	KChIP2		Long QT syndrome	[85,86]
			Cancer	
β-secretase precursor	BACE1	Cys474, 478, 482, 485	Alzheimer’s	[87,118]
G protein-coupled receptors	β2AR	Cys265		[100]
Ras proteins	p21-Ras	Cys188	Cancer	[107]

## Data Availability

Not applicable.
